# Comprehensive metabolic characterization of pediatric ependymomas

**DOI:** 10.1093/lifemeta/loag010

**Published:** 2026-04-20

**Authors:** Tong Li, Ying Jin, Sikang Ren, Yifan Liu, Dan Cheng, Zhanying Bi, Yanong Li, Xiaoli Chen, Xiaoqin Zhu, Zheng Chen, Weiwei He, Yangyang Li, Yuwei Liu, Guoming Luan, Yongji Tian, Yaou Liu, Woo-ping Ge

**Affiliations:** Beijing Institute for Brain Research, Chinese Academy of Medical Sciences & Peking Union Medical College, Beijing 102206, China; Chinese Institute for Brain Research, Beijing 102206, China; Chinese Institute for Brain Research, Beijing 102206, China; Department of Radiology, Beijing Tiantan Hospital, Capital Medical University, Beijing 100070, China; Department of Neurosurgery, Beijing Tiantan Hospital, Capital Medical University, Beijing 100070, China; Department of Neurosurgery, Beijing Tiantan Hospital, Capital Medical University, Beijing 100070, China; Department of Radiology, Beijing Tiantan Hospital, Capital Medical University, Beijing 100070, China; Chinese Institute for Brain Research, Beijing 102206, China; College of Life Sciences, Nankai University, Tianjin 300071, China; Chinese Institute for Brain Research, Beijing 102206, China; Department of Radiology, Beijing Tiantan Hospital, Capital Medical University, Beijing 100070, China; Chinese Institute for Brain Research, Beijing 102206, China; Institute of Biophysics, Chinese Academy of Sciences, Beijing 100101, China; Changping Laboratory, Beijing 102206, China; Chinese Institute for Brain Research, Beijing 102206, China; Department of Radiology, Beijing Tiantan Hospital, Capital Medical University, Beijing 100070, China; Chinese Institute for Brain Research, Beijing 102206, China; Department of Neurosurgery, Sanbo Brain Hospital, Capital Medical University, Beijing 100093, China; Chinese Institute for Brain Research, Beijing 102206, China; Department of Basic Medical Science, Capital Medical University, Beijing 100069, China; Department of Radiology, Beijing Tiantan Hospital, Capital Medical University, Beijing 100070, China; Chinese Institute for Brain Research, Beijing 102206, China; Department of Radiology, Beijing Tiantan Hospital, Capital Medical University, Beijing 100070, China; Department of Neurosurgery, Sanbo Brain Hospital, Capital Medical University, Beijing 100093, China; Department of Neurosurgery, Beijing Tiantan Hospital, Capital Medical University, Beijing 100070, China; Department of Radiology, Beijing Tiantan Hospital, Capital Medical University, Beijing 100070, China; Beijing Institute for Brain Research, Chinese Academy of Medical Sciences & Peking Union Medical College, Beijing 102206, China; Chinese Institute for Brain Research, Beijing 102206, China; Changping Laboratory, Beijing 102206, China

## Abstract

**Graphical Abstract:**
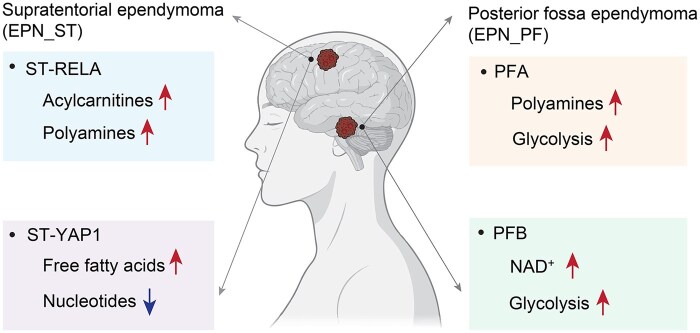

Dear Editor,

Ependymoma is a neuroepithelial neoplasia of the central nervous system that occurs primarily in children but also can occur in adults. Regardless of patient age, ependymoma can arise throughout the neuroaxis, including the supratentorial region (ST), posterior fossa (PF), and spinal cord [1]. In children, it represents the third most common brain tumor, with approximately 90% of cases occurring intracranially—two-thirds in the PF and one-third in the ST [2]. Chemotherapy has shown limited efficacy for most pediatric patients, leaving surgical resection with subsequent radiotherapy as the mainstay of treatment. Consequently, up to 40% of ependymomas remain incurable [1].

Over the past two decades, the understanding of ependymoma has expanded from purely histopathological classification to molecular subtyping, which has greatly advanced insights into the disease and improved the prediction of prognosis [1–5]. This progress has been largely driven by results obtained from whole-exome sequencing, single-cell transcriptomics [4, 5], and integrative epigenomic analyses [2, 3], which have uncovered numerous oncogenic drivers and molecularly defined subtypes. Supratentorial ependymomas (EPN_ST) are primarily divided into *ZFTA*–*RELA* fusion–positive EPN_ST (EPN_ST-RELA [ST-RELA]), characterized by the *ZFTA–RELA* fusion gene resulting from a chromosomal rearrangement, and YAP1 fusion–positive EPN_ST (EPN_ST-YAP1 [ST-YAP1]), defined by *YAP1* gene rearrangements or fusions [2, 3]. Among these, ST-YAP1 is associated with significantly better prognosis than is ST-RELA [3]. PF ependymomas (EPN_PF) are classified into EPN_PF group A (PFA) and EPN_PF group B (PFB) based on DNA methylation profiling, with PFA characterized by CpG island hypermethylation and loss of trimethylation of lysine 27 in histone H3 (H3K27me3) [1]. PFB carries a distinctly more favorable prognosis than PFA [3]. Despite the aforementioned discoveries of oncogenic drivers and molecular subtypes, the metabolic phenotypes distinguishing these subtypes remain largely unexplored. Furthermore, given that the incidence of ependymoma in children is only 3 per 1,000,000 [1], obtaining a sufficiently large cohort for a comprehensive study remains a major challenge.

Metabolomics provides a system-level snapshot of metabolites derived from tumor cells, brain cells, and their microenvironment [6, 7]. It has emerged as a powerful tool for tumor stratification, detection, and therapeutic intervention. Metabolic reprogramming not only sustains the bioenergetic and biosynthetic requirements of rapidly proliferating cells but also influences the epigenetic landscape, thereby driving tumor cell heterogeneity and evolution [8]. Nonetheless, the metabolic features that distinguish ependymoma subtypes—such as ST-RELA versus ST-YAP1 or PFA versus PFB—remain poorly understood. Therefore, defining subtype-specific metabolic signatures will be critical for identifying metabolic vulnerabilities and guiding precision therapeutic strategies tailored to the molecular context of each subtype.

Toward this goal, we conducted a comprehensive metabolomic analysis of 42 pediatric ependymomas encompassing the aforementioned four molecularly defined subtypes (Supplementary Table S1): ST-RELA (*n *= 11), ST-YAP1 (*n *= 7), PFA (*n *= 19), and PFB (*n *= 5) (Fig. 1a; Supplementary Fig. S1a). We identified both the metabolic landscapes of these subtypes and their distinct metabolic signatures.

**Figure 1:**
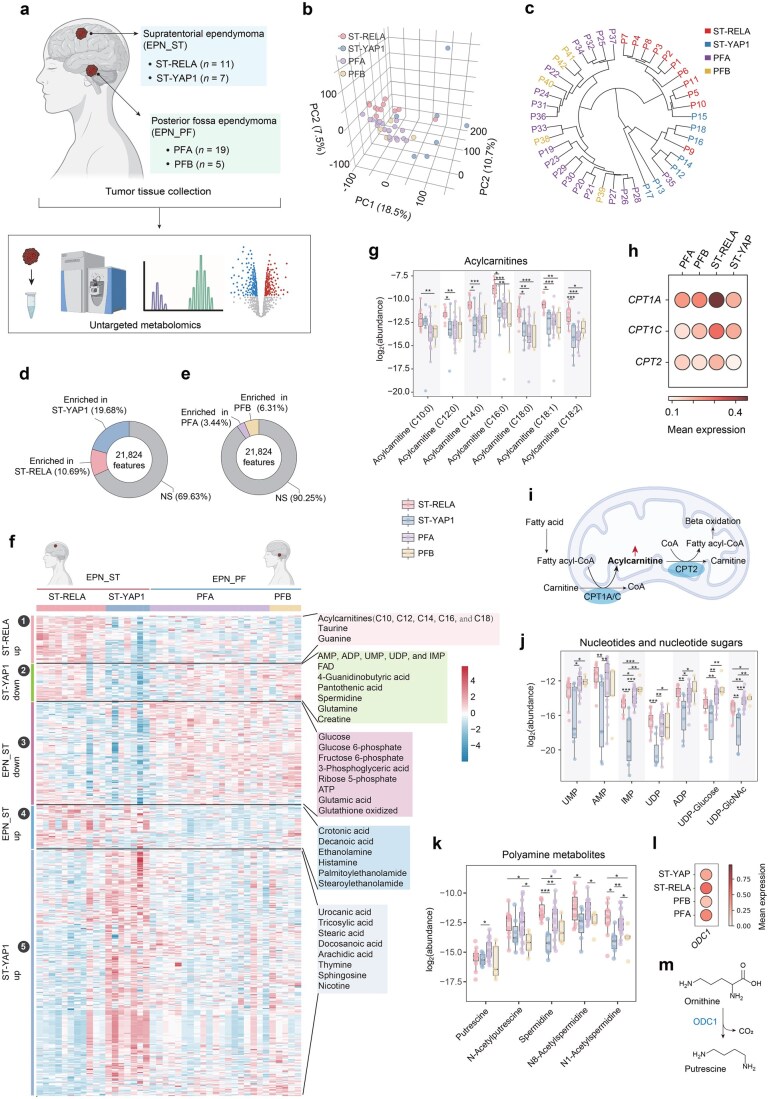
Metabolomic profiling of pediatric ependymoma subtypes. (a) Schematic illustration showing tumor-tissue collection for untargeted metabolomic profiling. Schematic diagram was created with BioRender. (b) Three-dimensional principal component analysis (3D PCA) of all tissue samples. Points are colored according to molecular subtype, with principal components 1, 2, and 3 (PC1, PC2, and PC3) defining the axes. The percentages in parentheses indicate the proportion of total variance explained by each principal component. (c) Hierarchical clustering dendrogram of all tissue samples. Sample labels are colored according to their respective molecularly defined subtypes. Branch lengths represent the degree of similarity (or distance) between samples and clusters, with shorter branches indicating more similarity. (d) Proportional distribution plot illustrating the enrichment of differential meta­bolites in ST-RELA and ST-YAP1. NS, not significant. (e) Proportional distribution plot illustrating the enrichment of differential metabolites in PFA and PFB. NS, not significant. (f) Heatmap displaying the differential metabolites across the four ependymoma subtypes, revealing distinct hierarchical clustering patterns. From top to bottom, the major clusters can be summarized as: ST-RELA enriched (label 1); ST-YAP1 reduced (label 2); EPN_ST reduced (label 3); EPN_ST enriched (label 4); ST-YAP1 enriched (label 5). A significance threshold of *P *< 0.05 was used. (g, j, and k) Box plots showing the abundance of representative metabolites in ST-RELA, ST-YAP1, PFA, and PFB, including acylcarnitines (g), nucleotides and nucleotide sugars (j), and polyamine meta­bolites (k). ^*^P < 0.05; ^**^P < 0.01; ^***^P < 0.001. (h and l) Bubble plots showing the relative expression of *CPT1A*, *CPT1C*, *CPT2* (h) and *ODC1* (l) across the four ependymoma subtypes in dataset 2 [5], with the color intensity of each bubble indicating the mean expression level within each subtype. (i) Schematic illustration showing the transport of free fatty acids into the mitochondria via the action of the three enzymes CPT1A, CPT1C, and CPT2 for β-oxidation. The upward arrow indicates the enrichment of acylcarnitines in ST-RELA. (m) Schematic illustration showing the conversion of ornithine to putrescine catalyzed by ODC1.

To systematically investigate the metabolic characteristics of different pediatric ependymoma subtypes, we performed untargeted metabolomic profiling of all collected tumor tissue samples (Fig. 1a). Subtype classification was determined by experienced pathologists based on pathological and molecular features. Specifically, for EPN_ST, ST-RELA was defined by positive L1 cell adhesion molecule (L1CAM) immunohistochemistry together with sequencing evidence for a *ZFTA*–*RELA* gene fusion, whereas ST-YAP1 was defined by L1CAM negativity and the presence of a *YAP1* gene fusion. For EPN_PF, molecular subtyping was based on established methylation profiling criteria, which could distinguish PFA (characterized by diminution of H3K27me3 and a DNA methylation profile consistent with PFA) from PFB (H3K27me3 retention and a DNA methylation profile consistent with PFB). Untargeted metabolomic profiling of these tumor tissues yielded 21,296 metabolic features for downstream analyses.

To compare the overall metabolic profiles of the four subtypes, we first performed unsupervised principal component analysis (PCA) on all detected metabolic features (Fig. 1b). ST-YAP1 exhibited a markedly distinct metabolic profile compared with the other three subtypes, representing the first observation of such a pronounced metabolic divergence. In the PCA plot (Fig. 1b), ST-RELA also segregated clearly from PFA and PFB, whereas PFA and PFB had highly similar metabolic profiles. Consistently, the use of hierarchical clustering enabled the separation of ST-RELA and ST-YAP1 from all EPN_PF with 100% sensitivity (18/18) and 96% specificity (23/24) (Fig. 1c). Notably, despite both arising from the ST, ST-RELA, and ST-YAP1 displayed the greatest metabolic divergence (Fig. 1b and c). These findings suggest that the metabolic profiles of ependymomas are not strictly determined by anatomical (tumor subdomain) location and highlight the pronounced metabolic differences between ependymoma subtypes.

To investigate the metabolic differences between ST-RELA and ST-YAP1, we performed systematic differential analysis. Unsupervised PCA revealed pronounced differences in overall metabolic profiles between these two subtypes (Supplementary Fig. S1b). A total of 6627 distinct metabolic features were identified (Fig. 1d), among which 2333 features were enriched in ST-RELA, representing 10.69% of all detected features (e.g., flavin adenine dinucleotide, spermidine, taurine, and hypotaurine; Supplementary Fig. S1c), whereas 4294 features were enriched in ST-YAP1, representing 19.68% of all features (e.g., cystine, nicotine, and pentadecanoate; Supplementary Fig. S1c).

We further investigated the metabolic distinctions between PFA and PFB. Unlike supratentorial gliomas, PFA and PFB had highly similar global metabolic profiles as assessed by PCA (Supplementary Fig. S1d). Indeed, the overall metabolic difference between PFA and PFB was markedly smaller than that observed between ST-RELA and ST-YAP1. Subsequent differential analysis identified 2130 significantly altered metabolic features (Fig. 1e; Supplementary Fig. S1e), among which 751 were enriched in PFA (3.44% of the total; e.g., homocarnosine and glycine betaine) and 1379 were enriched in PFB (6.31% of the total; e.g., nicotinamide adenine dinucleotide [NAD^+^] and N-acetylaspartylglutamic acid [NAAG]). We also found that the S-adenosylmethionine/S-adenosylhomocysteine (SAM/SAH) ratio was significantly larger in PFA than that in PFB (fold change = 2.2; Supplementary Fig. S1f). As the primary cellular methyl donor, SAM provides methyl groups for the methylation of DNA and proteins (including histones), producing SAH as a byproduct (Supplementary Fig. S1g). Thus, the SAM/SAH ratio reflects the cellular methylation potential. Clinically, the most distinctive features between PFA and PFB were found to be CpG island hypermethylation and a reduced level of H3K27me3 [1]. These findings suggest that the elevated SAM/SAH ratio may enhance the cellular methylation potential, contributing to the CpG island hypermethylation characteristic of PFA. Moreover, the results implied that the relative diminution of H3K27me3 in PFA was unlikely attributable to impaired methylation potential.

To further clarify which changes are specific to individual subtypes and which reflect metabolic differences between EPN_ST and EPN_PF, we performed a multi-group difference analysis. A heatmap of differential metabolites revealed hierarchical patterns, including both shared and distinct metabolic features among the different subtypes and tumor subdomain locations (Fig. 1f). *Post hoc* analyses revealed that ST-YAP1 and ST-RELA had the largest number of differential metabolic features (5834 features), followed by ST-YAP1 and PFA (5793 features). By contrast, the two most clinically aggressive subtypes, ST-RELA and PFA, differed in 3529 features, whereas PFA and PFB differed in only 713 features (Supplementary Fig. S1h). This indicated that, although both ST-RELA and ST-YAP1 originate from the ST, the metabolic differences between them are substantially greater than those between EPN_ST and EPN_PF.

Notably, ST-YAP1—the subtype associated with the most favorable prognosis—had a distinct metabolic profile, with the upregulated metabolites accounting for approximately half of all differential metabolites, particularly characterized by a marked enrichment of free fatty acids (Fig. 1f; Supplementary Fig. S1i). These results were further confirmed by matrix-assisted laser desorption/ionization mass spectrometry imaging (MALDI-MSI). We found that fatty acids were indeed enriched in YAP1 (Supplementary Fig. S1k), consistent with our measurements from liquid chromatography-mass spectrometry (LC-MS) (Supplementary Fig. S1i). In contrast, acylcarnitines were exclusively enriched in ST-RELA (Fig. 1g), underscoring how distinct lipid metabolic interactions may underlie the divergent clinical aggressiveness observed for these two supratentorial subtypes. As the more aggressive subtype, ST-RELA may rapidly convert intracellular free fatty acids into acylcarnitines for mitochondrial β-oxidation to sustain energy production, thereby supporting rapid tumor growth. Consistent with this, the end product of β-oxidation, namely acetyl coenzyme A, was significantly enriched in ST-RELA (Supplementary Fig. S1j). For validation, we analyzed the expression of enzymes related to fatty acid β-oxidation in two publicly available single-cell RNA-seq datasets of pediatric ependymomas [4, 5]. Both analyses revealed upregulation of carnitine palmitoyl transferase 1A (*CPT1A*), *CPT1C*, and *CPT2*—key genes mediating β-oxidation—in ST-RELA tumors (Fig. 1h and i; Supplementary Fig. S2a–e), further supporting the reprogramming of fatty acid metabolism in this subtype. Analysis of the Pediatric Brain Tumor Atlas dataset from PedcBioPortal [9], integrating transcriptomic and survival data, revealed that high *CPT1A* expression was associated with poorer overall survival in EPN (Supplementary Fig. S3a). In contrast, *CPT1A* expression showed no prognostic relevance in other common pediatric brain tumors, including high-grade gliomas, medulloblastomas, atypical teratoid/rhabdoid tumors, and diffuse intrinsic pontine gliomas, highlighting the unique relevance of *CPT1A* in EPN (Supplementary Fig. S3b).

To validate whether lipid metabolism is upregulated in the RELA subtype, we performed bulk RNA-seq on 38 EPN tumor samples. Multiple lipid metabolism–related genes were upregulated in the RELA subtype, including those involved in fatty acid synthesis (*FASN*), fatty acid β-oxidation (*CPT1A*), lipid storage (*DGAT1*, *PNPLA2*, and *LIPE*), membrane lipid remodeling (*LPCAT1*), and lipid buffering (*ACOT7*) (Supplementary Fig. S4b). Notably, *LPCAT1*, *DGAT1*, and *ACOT7* have also been reported as marker genes of the RELA subtype [3]. These results further demonstrate the upregulation of lipid metabolism in the RELA subtype.

To further define the cellular functions associated with acylcarnitine alterations, we performed Spearman correlation analysis between acylcarnitine levels and gene expression. For carnitine 18:0 (stearoylcarnitine), 586 genes were positively correlated and 558 genes were negatively correlated (|*r*| > 0.5, *P *< 0.05), followed by separate enrichment analyses. Positively correlated genes were enriched in glucose metabolism, AMP-activated protein kinase signaling, and lipid metabolism (Supplementary Fig. S4a). Lipid metabolism–related genes were primarily involved in lipid supply (e.g., *FASN* and *LIPE*), lipid modification (e.g., *SCD* and *ELOVL5*), and membrane lipid remodeling (e.g., *DGAT1*, *LPCAT3*, and *DHCR7*). In contrast, negatively correlated genes were enriched in processes such as chemotaxis (*PTK2B*, *HSPB1*, *KDR*, *IDO1*, *MCU*, etc.), vascular endothelial growth factor receptor (VEGF) signaling (*HSPB1*, *KDR*, *CAV1*, *CDH13*, *CCN3*, etc.), and epithelial cell–cell adhesion (*ITGB5*, *CCN3*, *PKP3*, *FOXA3*, and *EMCN*) (Supplementary Fig. S4c). These results suggest that elevated acylcarnitine levels may be associated with immune evasion, endothelial cell dysfunction, and reduced cell–cell adhesion.

Another important metabolic feature of ST-YAP1 was the selective lower levels of nucleotides and nucleotide sugars (Fig. 1j). Among these, AMP, ADP, UMP, UDP, and IMP are central metabolites of the purine and pyrimidine nucleotide network. UDP-glucose and UDP-N-acetylglucosamine are central donors for glycosyltransferase reactions required for glycogen and glycoprotein biosynthesis. Surprisingly, IMP and AMP were among the most significantly altered metabolites, with decreases of 23.15-fold and 110.79-fold in ST-YAP1 than ST-RELA, respectively (Supplementary Fig. S1c). This specific metabolic signature may reflect a reduced demand for nucleotide synthesis and glycosylation, indicating that these tumor cells may be in a low-proliferation or quiescent state, which aligns with the relatively mild clinical phenotype of ST-YAP1 [3, 5]. Consistently, correlation analysis revealed that IMP levels were positively associated with 53 cell cycle–related genes (Supplementary Fig. S4d), including those involved in G1/S entry (*CDC6*, *CDT1*, *MCM3*, and *MCM5*), DNA synthesis (*RRM2*, *DHFR*, and *PRIM2*), G2/M transition (*CCNB1*, *CDK1*, *AURKA*, and *PLK1*), and DNA repair (*RAD51*, *BRIP1*, *CLSPN*, and *PHNO1*). As IMP is a key intermediate in *de novo* purine synthesis and recycling, these positive correlations suggest that ST-RELA tumors adopt a high nucleotide flux–high cell cycle–driven pattern, whereas ST-YAP1 tumors may be less dependent on purine metabolism.

In addition, polyamines and acetylated polyamines, including putrescine, N-acetylputrescine, spermidine, N1-acetylspermidine, and N8-acetylspermidine, were enriched in both PFA and ST-RELA (Fig. 1k). Pathway-level mapping also revealed that the entire arginine and proline metabolism pathways (where polyamines are enriched) were markedly enriched (Supplementary Fig. S5a–c). Polyamines such as putrescine and spermidine promote cell proliferation and maintain stemness by supporting nucleic acid biosynthesis, chromatin remodeling, and translation initiation. Their accumulation has been reported across multiple tumor types and is mechanistically linked to tumor growth [10, 11]. The significant enrichment of polyamines in these subtypes, therefore, points to a shared metabolic vulnerability, which may underlie their clinical aggressiveness compared with PFB and ST-YAP1. Supporting this, we found that ornithine decarboxylase 1 (*ODC1*), which encodes the rate-limiting enzyme of polyamine biosynthesis, was specifically upregulated in PFA and ST-YAP1, indicating heightened polyamine biosynthesis in these two subtypes (Fig. 1l and m; Supplementary Fig. S2f). Correlation analysis further supported the functional roles of polyamines. For spermidine, 1308 genes were positively correlated and 1328 were negatively correlated (|*r*| > 0.5, *P *< 0.05). In the biological process of cytoplasmic translation, 79 out of 136 genes showed significant and specific positive correlations with tissue spermidine levels (Supplementary Fig. S5d), including ribosomal proteins (ribosomal protein large/small subunit [*RPL/RPS*] family) and translation initiation factors (translation initiation factor [*EIF*] family). This is consistent with the core function of polyamines in stabilizing RNA structure, maintaining ribosome integrity, and promoting translation initiation. In contrast, negatively correlated genes were enriched in cilium movement, with 104 of 277 genes significantly associated (Supplementary Fig. S5e), including key ciliary components (*DNAH11*, *CCDC39*, *RSPH1*, and *DNAAF1*). The downregulation of cilia-related genes in high-spermidine samples suggests a link to ependymoma dedifferentiation and a more proliferative, stem-like state.

Age is a prognostic risk factor in PFA, with younger patients (< 5 years) exhibiting poorer outcomes; however, this association remains controversial. To further investigate the metabolic basis of this association, we compared tumor metabolomes between PFA patients younger than 5 years and those aged 5 years or older. Strikingly, we found that polyamine metabolites, which were already significantly enriched in PFA tumors overall, were further elevated in the younger group (Supplementary Fig. S5f). Among the 28 annotated differential metabolites, putrescine, N-acetylputrescine, spermidine, and N1-acetylspermidine were present at significantly higher levels in younger patients relative to older subjects. These findings suggest that PFA tumors from younger patients may be more dependent on polyamine metabolism to sustain proliferation, offering a potential rationale for age-tailored therapeutic strategies. Collectively, these findings highlight subtype-specific metabolic distinctions, particularly in fatty acid oxidation, nucleotide biosynthesis, and polyamine metabolism.

We also identified certain metabolites that were associated with tumor subdomain location (Fig. 1f). Upstream glycolytic intermediates, including glucose, glucose 6-phosphate, fructose 6-phosphate, and 3-phosphoglyceric acid, were enriched exclusively in PFA and PFB, with lactic acid also showing an increasing trend (Supplementary Fig. S5g), suggesting enhanced glucose uptake and glycolytic flux. Moreover, intermediates of the pentose phosphate pathway, such as sedoheptulose 7-phosphate and ribose 5-phosphate, were also elevated in PFA and PFB (Supplementary Fig. S5h). These results suggested that PFA and PFB may rely more strongly on glucose-driven anabolic and redox-supporting pathways than the other subtypes. Upregulation of glycolysis and the pentose phosphate pathway in PFA has also been reported [12], and patients with relatively higher expression of glycolytic genes have significantly shorter overall survival [12], underscoring the clinical relevance of this metabolic phenotype. Conversely, ethanolamine and ethanolamine-derived lipids, such as palmitoylethanolamide and stearoylethanolamide, were specifically enriched in EPN_ST (Supplementary Fig. S5i), indicative of metabolic reprogramming of membrane lipids. Collectively, these results suggest that ependymoma metabolism is shaped by both molecular subtype and tumor subdomain.

Our comprehensive metabolomic profiling provides the first systematic delineation of metabolic heterogeneity across ependymoma subtypes. By linking distinct metabolic signatures to both molecular subtype and anatomical (brain) origin, our study not only offers metabolic insights into tumor biology but also highlights potential diagnostic biomarkers and therapeutic vulnerabilities.

Consistent with prior genomic and epigenomic findings that defined ependymoma heterogeneity [1, 3], our results demonstrate that metabolic programs are not uniform but instead subtype specific. We found that the enrichment of acylcarnitines is a hallmark metabolic feature of ST-RELA, potentially reflecting enhanced fatty acid metabolism. Consistently, our transcriptomic data showed that multiple lipid metabolism–related genes were upregulated in the RELA subtype. Among them, *CPT1A*, which encodes an enzyme mediating the transport of fatty acids into the mitochondria, was significantly associated with poorer prognosis in EPN patients. This metabolic phenotype may be intrinsically associated with its unique oncogenic mechanisms [3]. RELA is a central transcription factor within the nuclear factor-κB (NF-κB) pathway, and the ZFTA–RELA fusion drives constitutive nuclear localization of RELA [3], resulting in persistent activation of NF-κB, which contributes to alterations in lipid metabolism.

The unique metabolic signature of ST-YAP1 is the low levels of nucleotides and nucleotide sugars. The reduction in their abundance may indicate that purine and pyrimidine biosynthesis is downregulated in ST-YAP1, which is highly consistent with the low level of proliferation and numerous differentiation features of this subtype [3]. In the present study, correlation analysis further indicated that higher levels of nucleotides, such as IMP, were associated with increased expression of cell cycle–related genes. Previous studies have shown that undifferentiated/progenitor-like states are typically associated with the upregulation of purine and pyrimidine metabolism [13], whereas multiple single-cell transcriptomic studies have demonstrated that ST-YAP1 is in a highly differentiated state characterized by reduced stemness [3, 5]. Additionally, some studies have indicated that nucleotide sugar biosynthesis is upregulated in stem/progenitor cells [14, 15], whereas such activity is significantly downregulated in differentiated cells [15]. These findings further suggest that the metabolic profile of ST-YAP1 stems from its unique characteristics of increased differentiation capacity and reduced proliferation.

PFA, the more clinically aggressive subtype, was enriched for polyamines and acetylated polyamines, metabolites that promote cell proliferation and tumor progression through chromatin remodeling, nucleic acid biosynthesis, and translation regulation [10]. This observation is consistent with previous reports demonstrating elevated polyamine metabolism in PFA compared with EPN_ST [12]. Importantly, when EPN_ST were further stratified into ST-RELA and ST-YAP1, we found that polyamine metabolites were simultaneously enriched in PFA and ST-RELA, the two subtypes with the poorest prognosis. Consistent with this inference, we found that *ODC1*, the gene encoding the rate-limiting enzyme of polyamine biosynthesis, was significantly enriched in PFA and ST-RELA. Recent studies have shown that polyamine metabolism supports ependymoma cell survival under hypoxic conditions, and pharmacological blockade of polyamine biosynthesis markedly suppresses PFA growth in a hypoxic state [12]. An alternative explanation is that polyamines may contribute to the maintenance of cellular stemness in PFA and ST-RELA. Previous studies have shown that both PFA and ST-RELA contain relatively high proportions of undifferentiated/progenitor-like cells [5], and polyamines have been widely reported to be abundant in proliferating stem and cancer cells, where they support stem cell maintenance, and inhibition of polyamine biosynthesis promotes differentiation [11, 16]. Importantly, our data further showed that intratumoral spermidine levels were positively correlated with ribosomal proteins and translation initiation factors, while negatively correlated with cilium movement–related processes. This pattern suggests that polyamine accumulation is associated with enhanced translational activity to support proliferation, alongside a shift away from differentiated ependymal functions, as ependymal cells are normally ciliated and maintain cerebrospinal fluid flow through coordinated ciliary beating. These findings may explain why polyamine enrichment has emerged as a common metabolic feature of the most aggressive subtypes, highlighting a potential therapeutic vulnerability.

## Limitations of the study

This study has several limitations. First, although our findings suggest subtype-specific metabolic dependencies in pediatric ependymomas, whether these metabolic vulnerabilities are therapeutically targetable requires further experimental validation. Second, the relatively limited sample size may constrain the robustness and generalizability of the identified metabolic characteristics. Future studies with larger cohorts and integrative multi-omics analyses are therefore needed to confirm and extend these findings. Finally, given that patients with ependymoma typically exhibit a median survival exceeding 10 years, we were unable to assess the association between metabolite profiles and survival outcomes. Long-term follow-up will be necessary to identify potential high-risk metabolic markers in pediatric ependymomas.

## Supplementary Material

loag010_Supplementary_Data

## Data Availability

The data that support the ﬁndings of this study are available in the article ﬁle. Supplementary information is available from the corresponding authors. An interactive website is available on which the data can be visualized.
