# Supplementary material for: Comprehensive metabolic characterization of pediatric ependymomas
Source: Life Metab. 2026 Apr 20;5(4):loag010. doi: 10.1093/lifemeta/loag010 (PMC13228137; doi:10.1093/lifemeta/loag010)
Supplement: loag010_Supplementary_Data [file loag010_supplementary_data.zip › Supplementary_data_0415-tu-Clear.docx]

**Supplementary Materials for**

**Comprehensive metabolic characterization of pediatric ependymomas**

Tong Li^1,2^, Ying Jin^2,3^, Sikang Ren^4^, Yifan Liu^4^, Dan Cheng^3^, Zhanying Bi^2,5^, Yanong Li^2,3^, Xiaoli Chen^2,6,7^, Xiaoqin Zhu^2,3^, Zheng Chen^2,8^, Weiwei He^2,9^, Yangyang Li^3^, Yuwei Liu^2,3^, Guoming Luan^8^, Yongji Tian^4,*^, Yaou Liu^3,*^, Woo-ping Ge^1, 2, 7, 10,*^

^1^Beijing Institute for Brain Research, Chinese Academy of Medical Sciences & Peking Union Medical College, Beijing 102206, China

^2^Chinese Institute for Brain Research, Beijing 102206, China

^3^Department of Radiology, Beijing Tiantan Hospital, Capital Medical University, Beijing 100070, China

^4^Department of Neurosurgery, Beijing Tiantan Hospital, Capital Medical University, Beijing 100070, China

^5^College of Life Sciences, Nankai University, Tianjin 300071, China

^6^Institute of Biophysics, Chinese Academy of Sciences, Beijing 100101, China

^7^Changping Laboratory, Beijing 102206, China

^8^Department of Neurosurgery, Sanbo Brain Hospital, Capital Medical University, Beijing 100093, China

^9^Department of Basic Medical Science, Capital Medical University, Beijing 100069, China

^*^Corresponding authors. Beijing Institute for Brain Research, Chinese Academy of Medical Sciences & Peking Union Medical College, Beijing 102206, China. E-mail: [woopingge@cibr.ac.cn](mailto:woo-ping.ge@utsouthwestern.edu) (W.p.G.); Department of Radiology, Beijing Tiantan Hospital, Capital Medical University, Beijing 100070, China. E-mail: [liuyaou@bjtth.org](mailto:liuyaou@bjtth.org) (Y.L.); Department of Neurosurgery, Beijing Tiantan Hospital, Capital Medical University, Beijing 100070, China. E-mail: tianyongji@bjtth.org

**Materials and methods**

**Tissue sample collection**

Tumor tissues were collected from pediatric ependymoma patients who underwent surgical resection at Beijing Tiantan Hospital, Capital Medical University, between 2016 and 2025. Molecular subtype classification was determined by experienced pathologists based on a comprehensive assessment of immunohistochemistry, next-generation sequencing, and DNA methylation profiling. Tumor specimens were frozen in liquid nitrogen within 5−30 min after surgical removal and stored at −80°C. This study was reviewed and approved by the Ethics Committee of Beijing Tiantan Hospital, Capital Medical University (approval number: KY2022-078-04). All patients signed an informed consent form.

**Metabolite extraction for LC-MS analysis**

Tumor tissues stored at −80°C were first equilibrated at −20°C for 30 min, and 20−30 mg of representative tissues were dissected for extraction. Each sample was accurately weighed, and pre-chilled 80% acetonitrile (80%:20% acetonitrile/H_2_O solution) was added at a ratio of 16 µL/mg of tissue to extract metabolites. The tissue was homogenized in 1.5-mL Eppendorf tubes using a grinder on ice. The resulting precipitates were resuspended by vortex and then pelleted by centrifugation at 21,130 *g* for 15 min at 4°C; the supernatants were collected and subjected to a second centrifugation under the same conditions. Clarified supernatants (100 µL) were then dried under vacuum using a SpeedVac concentrator (Thermo Fisher Scientific). For reconstitution, 60 µL of 80% acetonitrile was added to each dried extract. Samples were mixed for 30 min to ensure complete resuspension, followed by centrifugation at 21,130 *g* for 15 min at 4°C. Each resulting supernatant was transferred to an HPLC vial for LC-MS analysis.

**Untargeted metabolomics analysis**

Untargeted metabolomic profiling was performed as reported^[1,2]^ using a Thermo Fisher Vanquish liquid chromatography system coupled to a high-resolution Q Exactive mass spectrometer (Thermo Fisher Scientific, USA). Data were acquired in both negative electrospray ionization (ESI−) and positive (ESI+) modes^[3]^. Chromatographic separation was achieved using an Agilent Infinity Lab Poroshell 120 HILIC-Z column (2.1 × 100 mm, 2.7 μm) with PEEK lining.

For ESI+, mobile phase A consisted of 10 mmol/L ammonium formate in water, and mobile phase B was 10 mmol/L ammonium formate in acetonitrile. For ESI−, mobile phase A consisted of 10 mmol/L ammonium acetate in water, and mobile phase B was10 mmol/L ammonium acetate in acetonitrile. In both modes, HILIC separation was performed under the following gradient program: 0−4 min, 100% to 84% B; 4−11 min, 84% to 40% B; 11−12 min, held at 40% B; 12−13 min, 40% to 100% B; 13−17 min, re-equilibration at 100% B. The flow rate was 0.4 mL/min, and the injection volume was 3 μL. To monitor system stability, pooled quality-control samples, prepared by combining 20 μL aliquots from each biological sample, were injected at regular intervals (every 10 sample injections).

The MS parameters were as follows: spray voltage, 3.5 kV for ESI+ and 3.2 kV for ESI−; auxiliary gas flow rate, 10 arbitrary units; sheath gas flow rate, 30 arbitrary units; sweep gas flow rate, 5 arbitrary units; capillary temperature, 320°C; auxiliary gas heater temperature, 350°C; S-lens RF level, 55. For the MS1 method, the resolution was 70,000 in full-scan mode, automatic gain control (AGC) target was 3,000,000, maximum injection time was 100 ms, and the scan range was 60−900 m/z. For the MS2 method, the resolution was 17,500, AGC target was 50,000, minimum AGC target was set at 1,000, dynamic exclusion time was 6 s, maximum injection time was 80 ms, and the scan ranges were 60−400 m/z and 350−900 m/z. The normalized collision energy units were set at 20, 30, and 40.

**Metabolomics data processing and compound identification**

Raw LC-MS data were processed using Compound Discoverer 3.3 software (Thermo Fisher Scientific) for feature extraction, alignment, filtering, and annotation. Metabolic features were excluded if they met any of the following criteria: (ⅰ) signal-to-noise ratio < 3; (ⅱ) peak intensity < 30,000; or (ⅲ) sample-to-blank ratio < 3. The remaining features were annotated using both an in-house metabolite standard library comprising 462 well-characterized small molecules and several public databases, including mzCloud (mzcloud.org/), KEGG (kegg.jp/), HMDB (hmdb.ca/), MassBank (massbank.us/), Biocyc (biocyc.org/), and Lipid MAPS ([lipidmaps.org/](https://lipidmaps.org/)). The MS/MS data of the identified metabolites, including both positive and negative ion modes, are provided in Supplementary Table S2.

**Bulk RNA-seq**

Bulk RNA-seq was performed as previously described^[4]^**.** Briefly, total RNA was isolated from tumor tissues using the RNeasy Micro Kit (Qiagen). For library preparation, 1 μg of RNA per sample was used as input. Sequencing libraries were constructed with the Hieff NGS Ultima Dual-mode mRNA Library Prep Kit for Illumina (Yeasen Biotechnology) and subsequently sequenced on an Illumina NovaSeq platform. Raw sequencing reads in FASTQ format were subjected to quality control procedures, including the removal of adapter sequences, reads containing poly-N regions, and low-quality reads. Quality metrics such as Q20, Q30, GC content, and sequence duplication levels were then assessed based on the filtered clean data. All subsequent analyses were performed using these high-quality reads. The processed reads were aligned to the reference genome using HISAT2. Reads with no more than one mismatch were retained for downstream analyses and annotated according to the reference genome. Differential gene expression analysis between groups was conducted using DESeq2.

**MALDI-MSI**

For matrix-assisted laser desorption/ionization mass spectrometry imaging (MALDI-MSI), tumor tissues stored at −80°C were first equilibrated at −20°C prior to sectioning and subsequently cryosectioned into 12-μm thick slices using a Leica CM3050S cryostat. Tissue sections were mounted onto indium tin oxide (ITO)-coated glass slides (IING WEI) and digitized at 4800 dpi. Prior to matrix application, sections were dried, followed by uniform coating using an HTX TM-Sprayer (TMSP-M3, HTX Technologies). The matrix solution consisted of 5 mg/mL 2,5-dimethoxycinnamic acid (DMCA) prepared in 70% methanol with 0.1% trifluoroacetic acid. Spraying was performed at 75°C with a flow rate of 0.03 mL/min, nozzle velocity of 1,000 mm/min, track spacing of 3 mm, nebulizing pressure of 10 psi, and gas flow rate of 2 L/min. A total of eight passes were applied, with an interval of 3 s between consecutive layers to allow drying.

Following external calibration using red phosphorus, MSI data were acquired on a rapifleX MALDI-TOF/TOF mass spectrometer (Bruker Daltonics) equipped with a 10 kHz Smartbeam 3D laser system. Data were collected in negative ion mode under the following conditions: laser power at 50% (offset 12%), Smartbeam setting M5, ion source voltage 20.000 kV, lens voltage 11.350 kV, post-ion extraction 2.610 kV, reflector voltages of 20.850 kV (reflector 1), 1.085 kV (reflector 2), and 8.600 kV (reflector 3), with reflector gain set to 3.0×. Spectra were acquired over an m/z range of 90−400 with a digitizer rate of 2.5 GS/s. Imaging was performed at a spatial resolution of 100 μm, with 100 laser shots accumulated per pixel. Raw spectral data were processed and visualized using SCiLS Lab (version 2023c, Bruker Daltonics) and normalized based on total ion current (TIC).

**Statistical analysis**

The intensity of each metabolite feature was normalized to the total peak area of all metabolites within each sample, followed by log_2_ transformation. PCA was conducted on all metabolic features to assess global metabolic profiles across samples. For identifying differential metabolites between two groups (ST-RELA versus. ST-YAP1 or PFA versus PFB), the Student’s *t*-test (two-sided) was applied when both normality and homogeneity of variance were satisfied, Welch’s *t*-test (two-sided) was used when normality held but variances were unequal, and the Mann–Whitney *U* test (two-sided) was applied when at least one group deviated from normality. A significance threshold of *P* < 0.05 was used. For comparisons across all four groups, one-way analysis of variance was performed when all groups met the normality criterion, whereas the nonparametric Kruskal-Wallis rank-sum test was applied when any group failed to meet normality or had insufficient sample size for reliable analysis. Spearman correlation analysis was performed to assess the association between metabolite levels and gene expression, with a significance threshold set at *P* < 0.05.

**References**

1 Wang Y, Zhou L, Wang N *et al.* Comprehensive characterization of metabolic consumption and production by the human brain. *Neuron* 2025;**113**:1708-22.e5.

2 He W, Yu J, Sun Y *et al*. Macrophages in noise-exposed cochlea: changes, regulation and the potential role. *Aging Dis* 2020;**11**:191-9.

3 Xiong N, Gao X, Zhao H *et al.* Using arterial-venous analysis to characterize cancer metabolic consumption in patients. *Nat Commun* 2020;**11**:3169.

4 Wang Y, Liang Y, Ai D *et al.* ADAM10 mediates macroglial cell fate decisions in the developing brain. *bioRxiv* 2023. Doi: 10.1101/2023.02.11.527059.

5 Gillen AE, Riemondy KA, Amani V *et al.* Single-cell RNA sequencing of childhood ependymoma reveals neoplastic cell subpopulations that impact molecular classification and etiology. *Cell Rep* 2020;**32**:108023.

6 Gojo J, Englinger B, Jiang L *et al.* Single-cell RNA-seq reveals cellular hierarchies and impaired developmental trajectories in pediatric ependymoma. *Cancer Cell* 2020;**38**:44-59.e9.

**Legends for Supplementary Figures**

**Supplementary Figure S1** Subtype-specific metabolic alterations within the same anatomical compartment. **(a)** Summary of patient information and clinical characteristics, including molecular group, sex, and age. (**b)** Principal component analysis (PCA) of all supratentorial ependymomas, including ST-RELA (red) and ST-YAP1 (blue). The confidence ellipse for each subtype denotes the 95% confidence boundary. (**c)** Volcano plots displaying the differential metabolic features between ST-RELA (right, red) and ST-YAP1 (left, blue). A significance threshold of *P* < 0.05 was used. 3-IAA, indole-3-acetic acid; FAD, flavin adenine dinucleotide. (**d)** PCA of all posterior fossa ependymomas, including PFA (purple) and PFB (yellow). The confidence ellipse for each subtype denotes the 95% confidence boundary. (**e)** Volcano plots displaying the differential metabolic features enriched in PFA (purple) and PFB (yellow). NANA, N-acetylneuraminic acid; DHEA sulfate, dehydroepiandrosterone sulfate; NAAG, N-acetylaspartylglutamic acid; NAD^+^, nicotinamide adenine dinucleotide. (**f)** Box plot showing the SAM/SAH ratio in PFA and PFB. ^*^*P* < 0.05. (**g)** Schematic illustration showing that SAM serves as a methyl donor for DNA methylation. (**h)** Post-hoc analysis showing the number of significantly differential metabolites between each pair of subtypes. The width of the gray connecting lines represents the abundance of differential metabolites, whereas the four bubbles denote the four molecular subtypes. (**i and j)** Box plots showing the abundance of fatty acids (i) and acetyl coenzyme A (acetyl-CoA) (j) in ST-RELA (red), ST-YAP1 (blue), PFA (purple), and PFB (yellow). ^*^*P* < 0.05; ^**^*P* < 0.01; ^***^*P* < 0.001. (**k)** MALDI-MSI showing the intratumoral spatial distribution of representative free fatty acids. The left panel corresponds to an ST-RELA sample, and the right panel to an ST-YAP1 sample; both were mounted on the same slide. MALDI-MSI signal intensity was scaled from 0% to 100%, with a spatial resolution of 100 μm. Scale bar: 4 mm.

**Supplementary Figure S2** Expression differences of genes related to fatty acid β-oxidation and polyamine biosynthesis among the four ependymoma subtypes. **(a and d)** UMAP (Uniform Manifold Approximation and Projection) dimensionality reduction of single-cell RNA-seq data, depicting cell-type classification and expression levels of *CPT1A*, *CPT1C*, and *CPT2* in two public datasets, namely dataset 1^[5]^ (a) and dataset 2^[6]^ (d). The different colors represent distinct ependymoma subtypes. **(b and e)** Violin plots illustrating the relative expression of *CPT1A*, *CPT1C*, and *CPT2* across the four ependymoma subtypes in dataset 1 (b) and dataset 2 (e), with expression levels on the *y* axis represented as normalized counts. (**c)** The bubble plot displays the relative abundance of CPT1A, CPT1C, and CPT2 across the four ependymoma subtypes in dataset 1, with the color intensity of each bubble indicating the mean expression level within each subtype. **(f)** Violin plot displaying the relative expression of *ODC1* across the four ependymoma subtypes in dataset 2.

**Supplementary Figure S3** Association between CPT1A expression and survival in pediatric brain tumors. (a and b) Kaplan–Meier survival curves showing the prognostic impact of CPT1A expression in EPN (a) and non-EPN pediatric brain tumors (b). The blue line represents patients with CPT1A expression below the median, and the red line represents patients with CPT1A expression above the median. *P* values were calculated using the log-rank test.

**Supplementary Figure S4** Genes and cellular processes associated with acylcarnitine level in the RELA subtype. (a and c) Enrichment analysis of genes positively (a) and negatively (c) correlated with tissue acylcarnitine 18:0 abundance. *P* values were calculated using the hypergeometric test. Biological processes related to lipid metabolism are highlighted in red. (b) Lipid metabolism–related genes upregulated in the RELA subtype and their expression levels. All genes highlighted in red indicate upregulation, including *FASN*, *LPCAT1*, *ACOT7*, *CPT1A*, *DGAT1*, *PNPLA2*, and *LIPE*. The bar plot shows their expression levels across the four EPN subtypes. (d) Enrichment analysis of genes positively correlated with tissue IMP abundance. *P* values were calculated using the hypergeometric test.

**Supplementary Figure S5** Differences in polyamine metabolism between subtypes and tumor subdomain–associated metabolites. **(a)** Summary diagram of representative upregulated metabolites in PFA within the polyamine metabolic pathway. Upward purple arrows indicate the enrichment of polyamine metabolites in ST-RELA and PFA. **(b and c)** KEGG pathway enrichment analysis of the differential metabolites for PFA vs. PFB (b) and ST-RELA vs. ST-YAP1 (c). Bubble transparency represents the *P* value, and bubble size indicates the number of hits (differential metabolites) in each pathway. The *x* axis represents the pathway impact factor. *P* values were calculated using a hypergeometric test. **(d and e)** Enrichment analysis of genes positively (d) and negatively (e) correlated with tissue spermidine abundance. *P* values were calculated using the hypergeometric test. (**f)** Volcano plot showing differential metabolic features between PFA tumors from patients younger than 5 years (left, blue) and those aged 5 years or older (right, red). A significance threshold of *P* < 0.05 was used. (**g**–i) Box plots displaying the abundance of glycolysis metabolites (g), sedoheptulose 7-phosphate and ribose 5-phosphate (h), and ethanolamine (i) across the four ependymoma subtypes. ^*^*P* < 0.05; ^**^*P* < 0.01; ^***^*P* < 0.001. NS, not significant.

**Supplementary Table S1** Patient information for the pediatric ependymoma samples analyzed in this study.

| Sample | Age (years) | Gender | Group |
| --- | --- | --- | --- |
| E1  E2  E3  E4  E5  E6  E7  E8  E9  E10  E11  E12  E13  E14  E15  E16  E16  E18  E19  E20  E21  E22  E23  E24  E25  E26  E27  E28  E29  E30  E31  E32  E33  E34  E35  E36  E37  E38  E39  E40  E41 | 1  10  2  1  7  2  4  3  3  9  3  4  3  6  3  7  4  1  2  5  8  9  6  8  6  5  8  8  2  5  7  9  1  4  2  5  6  12  10  15  6 | Male  Female  Male  Male  Female  Male  Female  Male  Female  Male  Male  Male  Male  Male  Male  Female  Male  Female  Female  Male  Male  Male  Female  Female  Male  Female  Female  Male  Female  Male  Male  Male  Male  Male  Male  Male  Female  Female  Male  Male  Male | EPN_ST_RELA  EPN_ST_RELA  EPN_PFA  EPN_PFA  EPN_ST_YAP1  EPN_PFA  EPN_PFA  EPN_PFA  EPN_ST_RELA  EPN_ST_RELA  EPN_ST_RELA  EPN_ST_RELA  EPN_ST_RELA  EPN_PFA  EPN_PFA  EPN_PFA  EPN_PFA  EPN_ST_YAP1  EPN_PFA  EPN_PFA  EPN_PFA  EPN_PFA  EPN_ST_YAP1  EPN_ST_YAP1  EPN_ST_YAP1  EPN_ST_YAP1  EPN_ST_YAP1  EPN_PFA  EPN_PFA  EPN_PFA  EPN_ST_RELA  EPN_ST_RELA  EPN_ST_RELA  EPN_ST_RELA  EPN_PFA  EPN_PFA  EPN_PFB  EPN_PFB  EPN_PFB  EPN_PFB  EPN_PFA |
| E42 | 4 | Male | EPN_PFB |
