# Supplementary figures and images for: Comprehensive metabolic characterization of pediatric ependymomas
Source: Life Metab. 2026 Apr 20;5(4):loag010. doi: 10.1093/lifemeta/loag010 (PMC13228137; doi:10.1093/lifemeta/loag010)

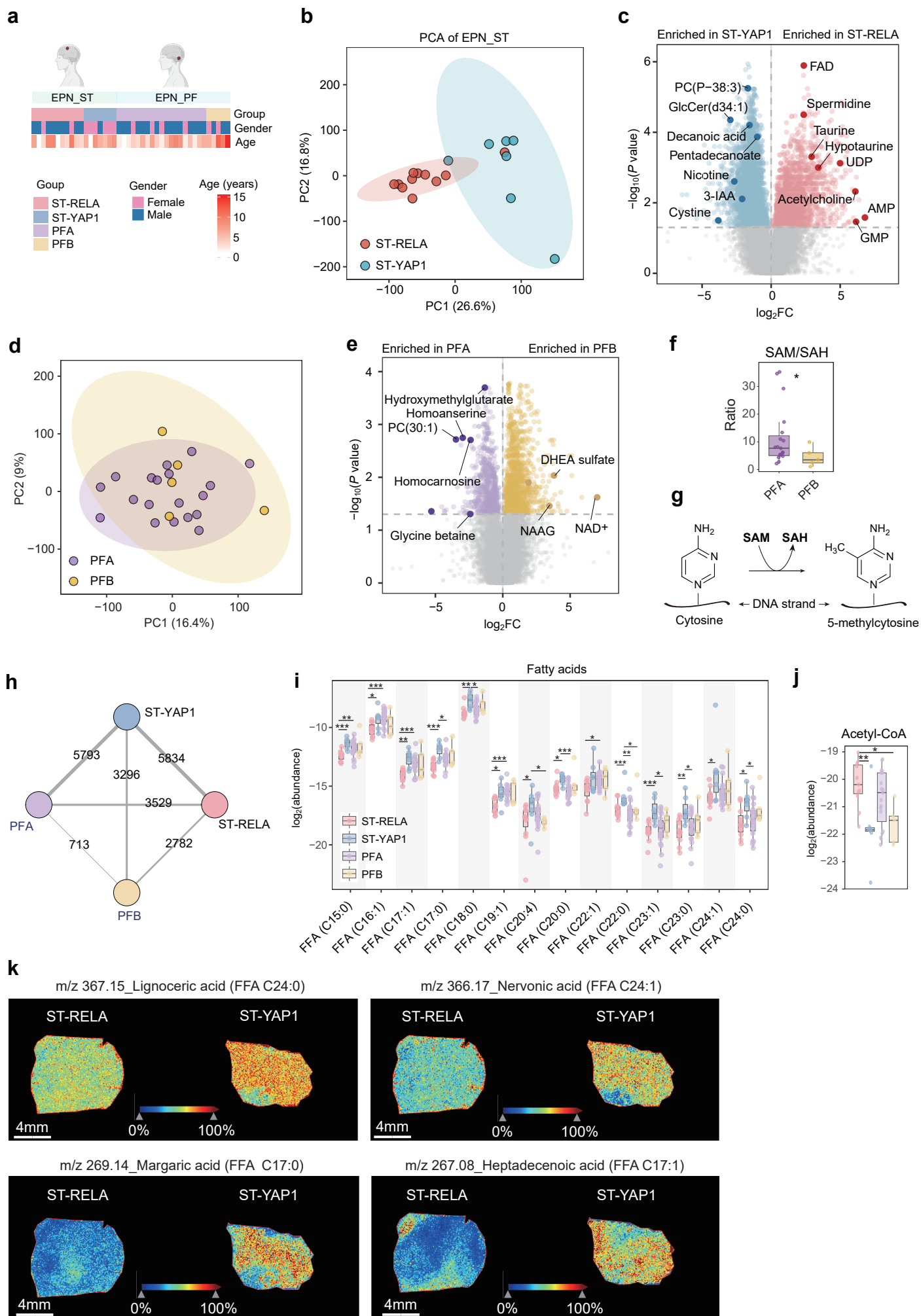

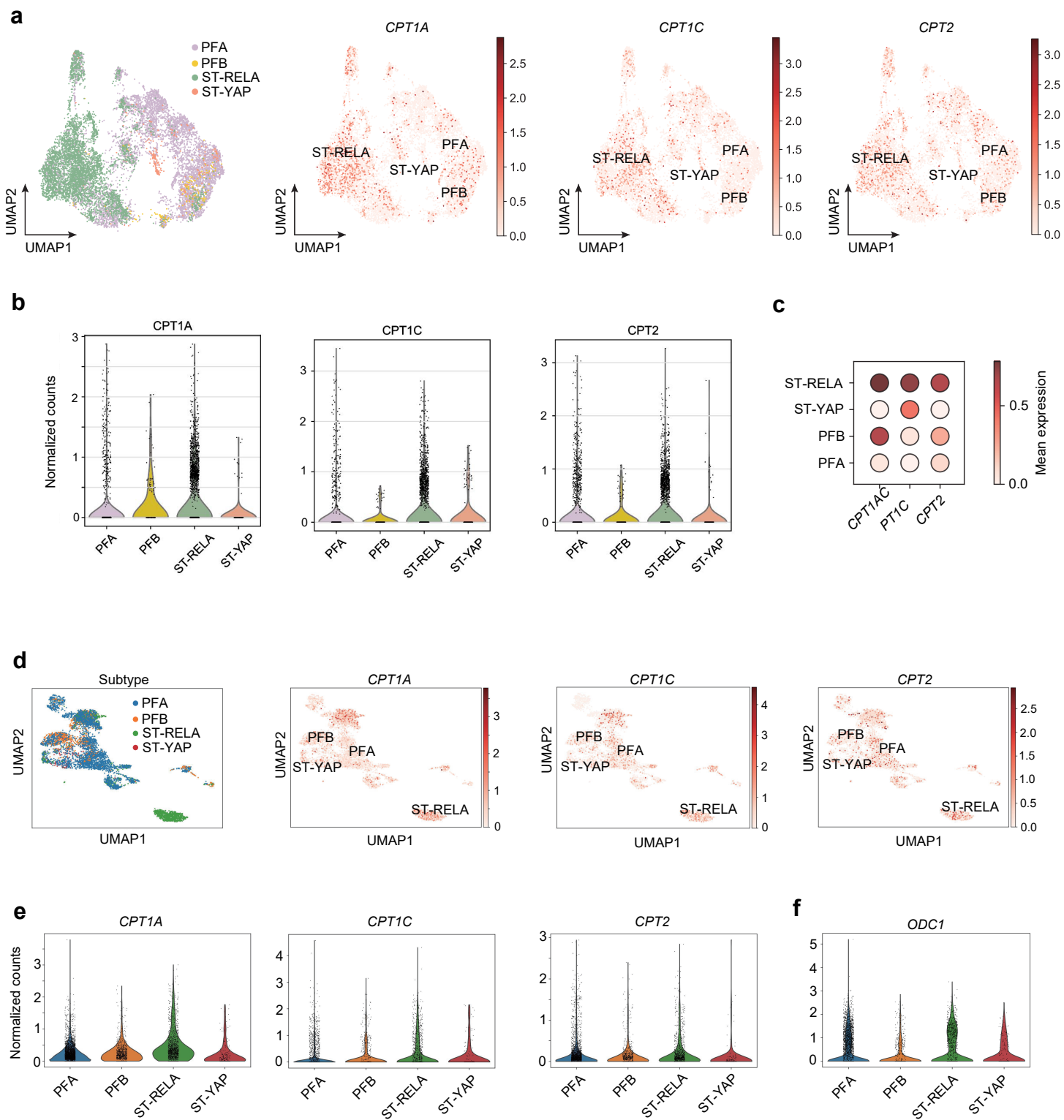

**a**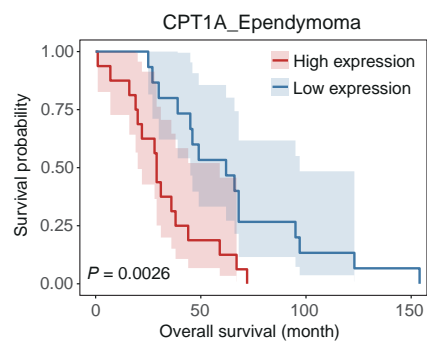**b**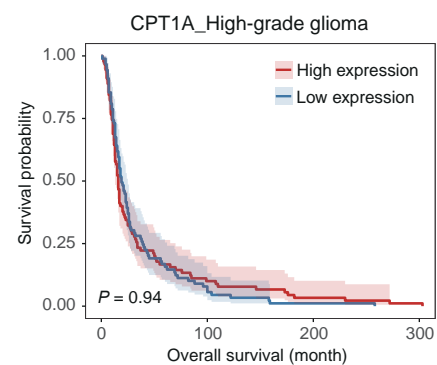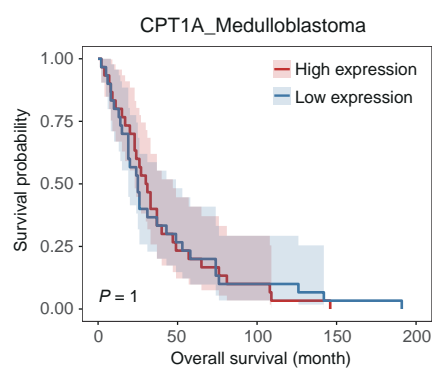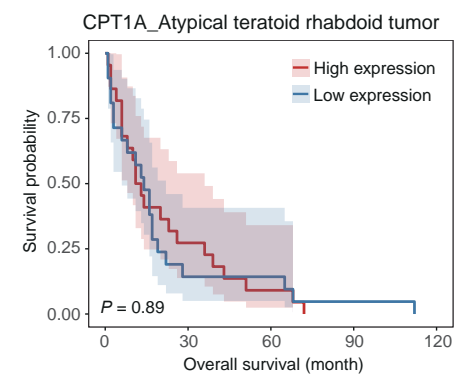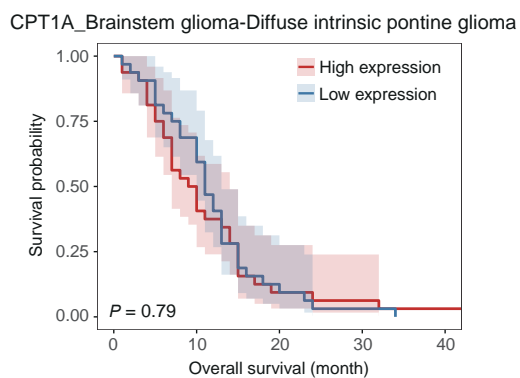

**a**

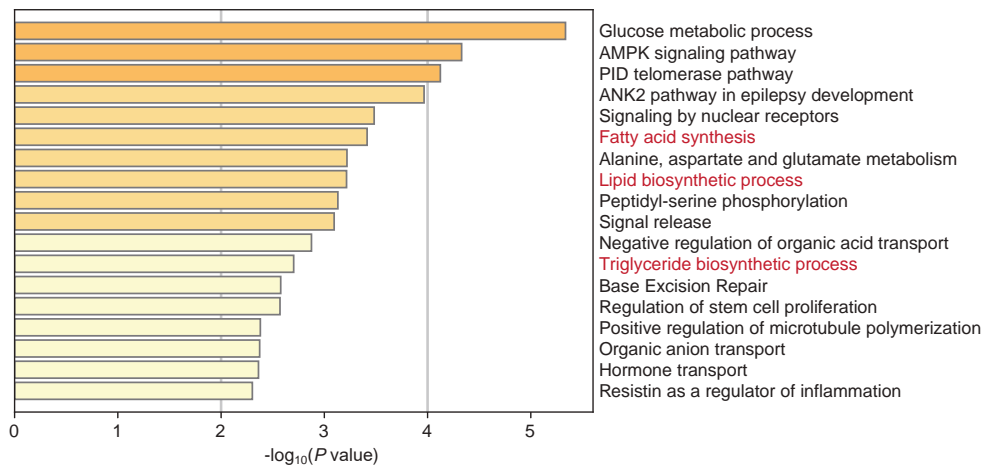

**b**

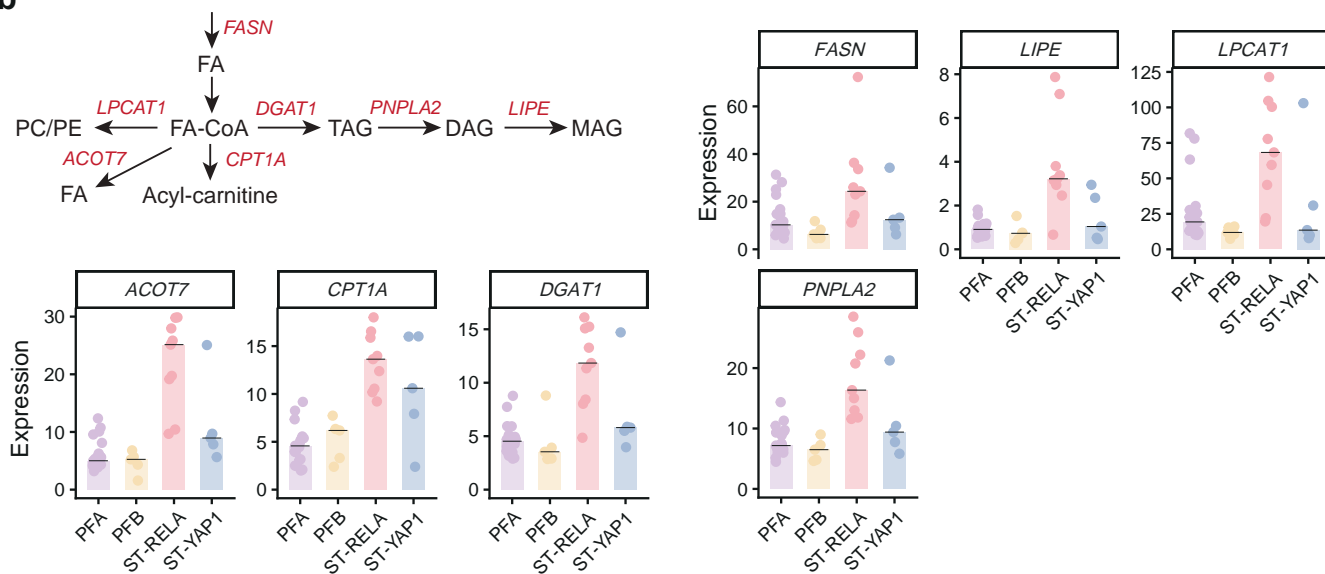

**c**

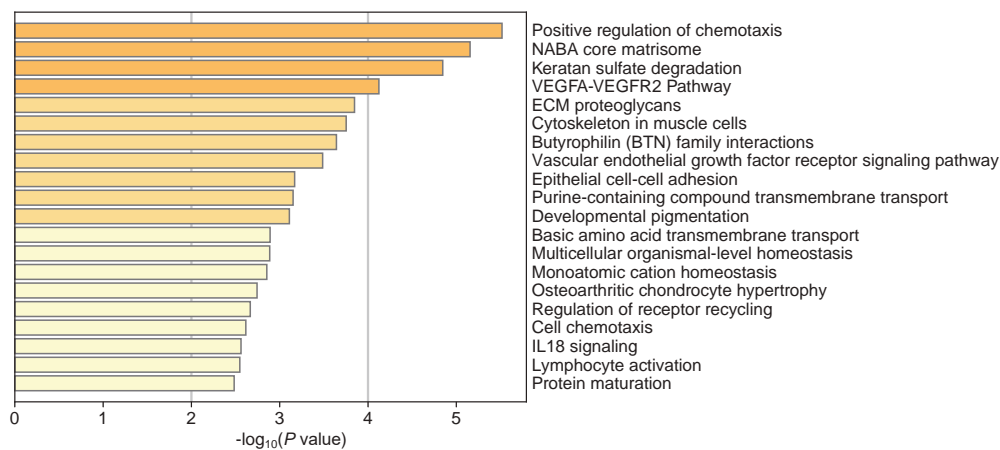

**d**

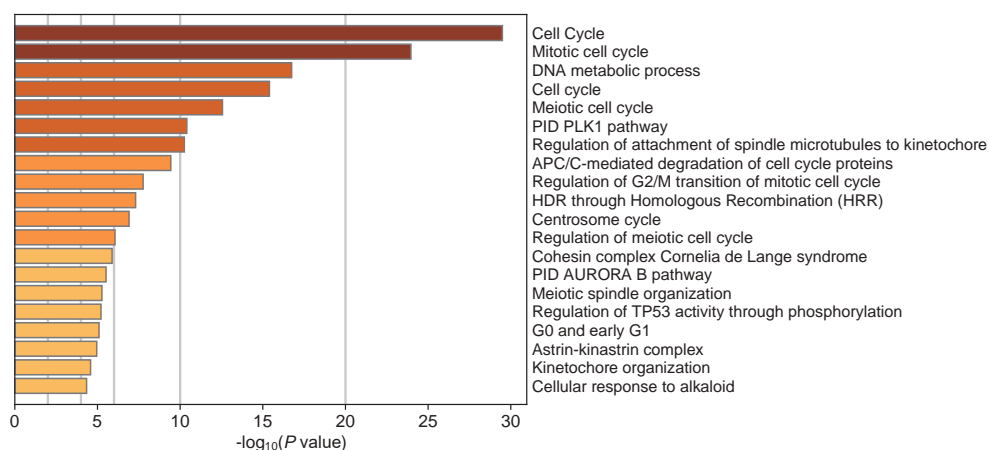

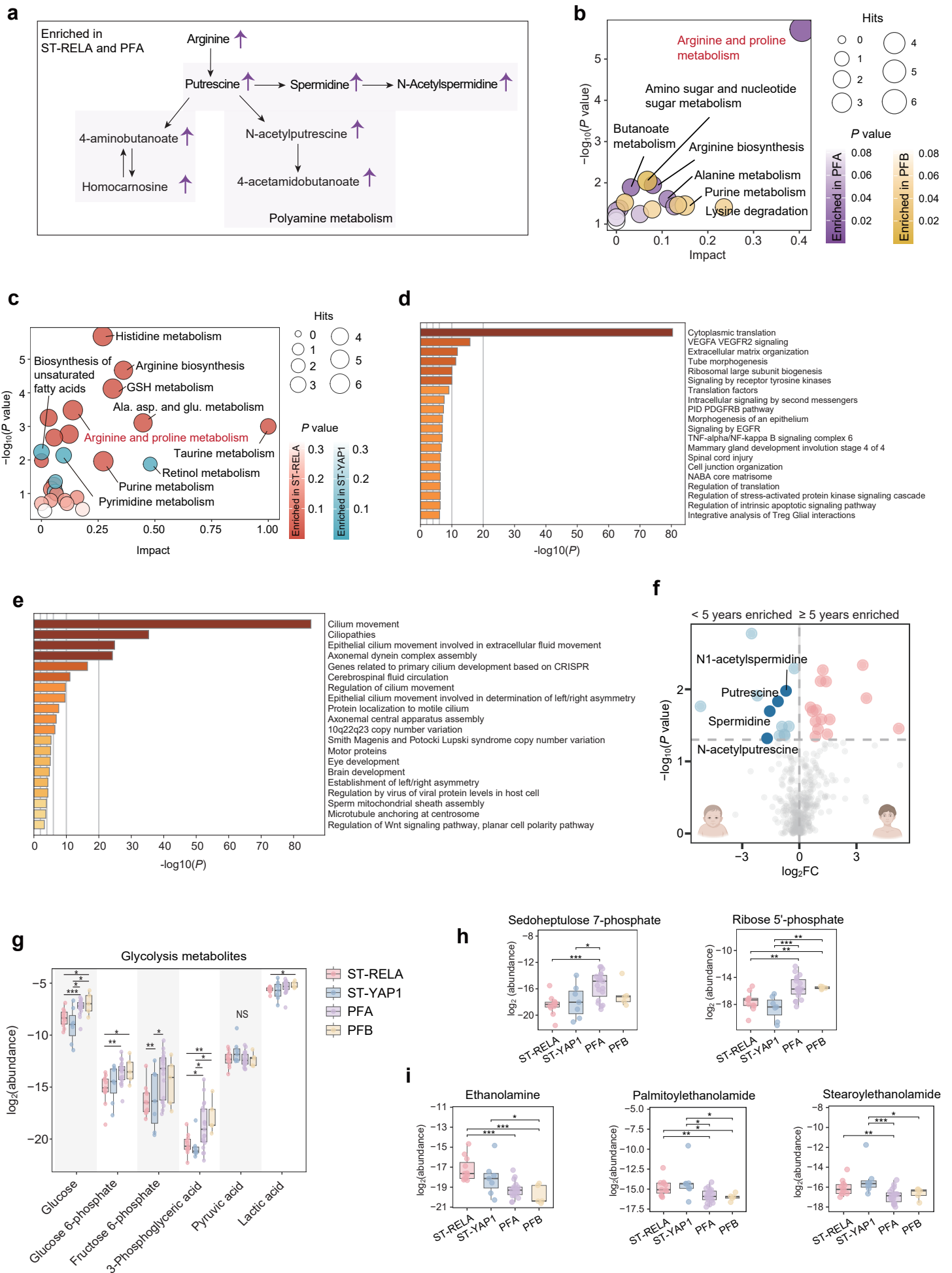

Supplement: loag010_Supplementary_Data [file loag010_supplementary_data.zip › Supplementary_figures.pdf]
